# A cell-based in vitro assay for testing of immunological integrity of Tetanus toxoid vaccine antigen

**DOI:** 10.1038/s41541-021-00344-1

**Published:** 2021-06-23

**Authors:** Olga Ticha, Dido Klemm, Lukas Moos, Isabelle Bekeredjian-Ding

**Affiliations:** Division of Microbiology, Paul-Ehrlich-Institut, Langen, Germany

**Keywords:** Inactivated vaccines, Protein vaccines, Bacterial infection

## Abstract

Vaccines containing inactivated toxins confer protection by eliciting a neutralizing antibody response against bacterial toxins such as tetanus and diphtheria. At present, release of tetanus toxoid (TT) and diphtheria toxoid (DT)-containing vaccines relies on in vivo experiments showing the protective vaccine response. The aim of this study was to develop a reliable in vitro assay for TT vaccine antigen characterization with the potential of replacing in vivo potency experiments. To this end, we exploited that TT elicits a recall response in vaccinated donors: human peripheral blood mononuclear cells (PBMC) were stimulated with alum-adsorbed TT bulk antigen and low concentrations of TLR9 ligand; induction of TT-specific IgG was quantified via ELISpot after 5 days. Proof-of-concept was obtained using paired samples from donors before and after vaccination; anti-TT IgG was only detected in PBMC collected after booster vaccination; specificity was demonstrated with DT stimulation as control. Notably, when using PBMC from buffy coats, the specific response to TT was reproducible in 30% of cells; responsiveness correlated with higher numbers of switched memory B cells. Consecutive results showed that TT-specific IgG was also detectable when PBMC were stimulated with DTaP final vaccine product. Thus, the assay provides a viable means to test B-cell differentiation and induction of TT-specific IgG secretion using bulk antigen and final vaccine. However, prequalification of PBMC is required for reliable performance. Along with physicochemical and immunochemical methods, the functional assay could represent a complementary tool to replace in vivo potency assays in batch release of TT-containing vaccines.

## Introduction

Vaccines confer safety and are the most effective means of protection against infectious disease as demonstrated by the success of vaccines against polio, smallpox, measles, diphtheria, and tetanus among others. Introduction of infant immunization against these pathogens or their disease-provoking toxins has led to an impressive reduction of global disease burden and improved survival of neonates and children worldwide.

Tetanus is a serious illness caused by exposure of a wound to the spores of the bacterium *Clostridium tetani*, which persist in soil, dust, saliva, or manure. The bacterial toxins affect the nervous system and infection leads to painful muscle spasms and ultimately to death. Tetanus toxoid (TT), e.g., tetanus toxin inactivated with formaldehyde, is the basis for tetanus vaccine formulations, which induce a strong and long-lasting neutralizing antibody response to the toxin^1–4^.

Booster vaccinations are required to acquire long-term protection against tetanus and to maintain immunity over a lifetime. Having established immunity in childhood, adolescents, and adults are recommended to receive booster vaccinations every 10 years at the latest^5,6^. Reflecting this, in Germany, >75% of adults have been vaccinated within last 10 years^3,7^.

TT-induced antibodies represent the correlate of protection for these vaccines, because they neutralize the bacterial toxin, and, thus, prevent disease manifestation. The antibodies are derived from the pool of long-lived plasma cells and memory B cells previously exposed to TT^8^. Anti-TT IgG serum antibody titers above 0.1 IU/ml are considered protective^6,9–11^.

The secondary immune response to antigen induces proliferation and differentiation of antigen-specific memory B cells into antibody-secreting cells, which is in vivo accompanied by a transient elevation in serum antibodies. This process has a key role in the neutralization and elimination of pathogens and their virulence factors^12–14^. The currently employed in vivo models for batch release testing of TT vaccines are based on inducing in vivo humoral immunity and evaluated either by protection from tetanus toxin challenge or presence of tetanus toxin-specific antibodies^15,16^.

In vivo potency assays used for consistency testing of vaccine batches were established decades ago. The need for replacement and reduction of in vivo methods for quality control of toxoid vaccines is well recognized and enforced by changes in the animal legislation including the European Convention for the Protection of Vertebrate Animals used for Experimental and other Scientific Purposes (ETS No. 123)^17^, Directive of the European parliament and of the council 2010/63/EU on the protection of animals used for scientific purposes^18^ or European Pharmacopoeia chapter (5.2.14) “Substitution of in vivo methods by in vitro methods for the quality control of vaccines”^19^. Subsequent method developments have focused on quantification of vaccine antigen, absence of toxin, and irreversibility of toxoid, the degree of adsorption of antigen to adjuvant in the final product as well as mass spectrometry-based analyses of protein content to detect inconsistencies in production and susceptibility to degradation^20–23^. However, to date, none of these methods can be used for potency testing of TT vaccines, which is classically associated with functional testing. In the present study, we describe a new assay that mimics the process of TT-induced specific antibody induction in vitro.

Although secretion of antibodies derived from activated plasmablasts can be quantified by seeding them on ELISpot membranes directly after isolation^24–26^, antigen-specific memory B cells need to be stimulated to undergo differentiation to antibody-secreting cells (ASC). Notably, a variety of protocols developed for detection and enumeration of vaccine and infection-induced antigen-specific memory B cells exist. They are usually based on 3–10-day cultures with addition of polyclonal stimuli, e.g., R848, CpG, pokeweed mitogen, *Staphylococcus aureus* Cowan strain I, CD40 ligand or anti-CD40 antibody, in combination with differentiation-supporting cytokines such as BAFF, IL-2, IL-6, and IL-10^12,24,27,28^. In contrast to previous approaches, the assay developed in this study avoids unspecific, polyclonal B-cell stimulation but makes use of the co-stimulatory potential of TLR9 ligand CpG to support antigen-driven terminal differentiation of memory B cells^29,30^.

Here, we present original data demonstrating that eliciting a recall response via dual stimulation with B-cell receptor (BCR) antigen and TLR9 ligand can be applied to confirm the presence and test the functional integrity of vaccine antigen in TT-containing vaccine batches.

## Results

### Proof-of-concept: stimulation of TT-specific IgG-secreting B cells in PBMC collected before and after vaccination

**Fig. 1:**
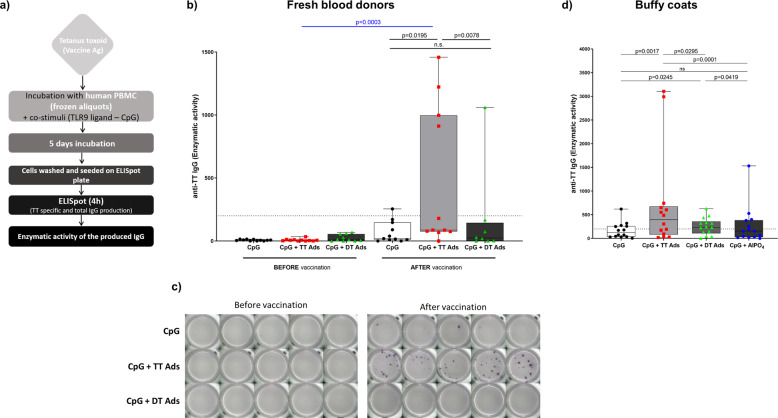
Proof-of-concept for a cell-based assay providing a functional readout for TT. **a** Assay layout. **b** Anti-TT IgG-specific enzymatic activity response in an assay performed with PBMC obtained from 11 donors before and after DTaP booster vaccination. Paired samples from individual donors before and after vaccination were tested in parallel. The graph summarizes the results from *n* = 3 independent experiments performed with 6, 4, and 1 donors, respectively. Bar graphs represents the interquartile range with median and whiskers depicting maximal and minimal values, each dot represents mean of enzymatic activity of a single donor. Data were analyzed using two-tailed Wilcoxon matched-pairs signed rank test (black lines and symbols), Mann–Whitney test (blue line and symbols). **c** ELISpot wells coated with TT for detection of anti-TT IgG of one representative donor showed (Donor #2). **d** Anti-TT IgG enzymatic activity of 14 buffy coat donors in response to CpG + adsorbed TT compared to the response to CpG + adsorbed DT or to CpG + aluminum adjuvant. Bar graphs represent the interquartile range with median and whiskers depicting maximal and minimal value, each dot represents mean of enzymatic activity of a single donor. Combined values from 14 buffy coats (*n* = 4 independent experiments) donors with statistical evaluation (Wilcoxon matched-pairs rank test). *P* values are depicted in the respective graphs, ns non-significant. The dotted line shows anti-TT IgG enzymatic activity of 200, the threshold of reactivity.

To enable in vitro testing of the functional integrity of TT vaccine antigen, we established a human PBMC assay based on dual stimulation of B cells with TT bulk antigen and a low concentration of TLR9 ligand CpG ODN 2006. The assay principle is summarized in Fig. 1a. In brief, a TT-specific B-cell response was triggered with TT, co-stimulation with B-cell-active CpG was included to support differentiation of B cells into ASC. TT-specific and total IgG were quantified via ELISPOT.

PBMC from healthy volunteers were obtained before and after vaccination with a booster dose of DTaP vaccine. Cells obtained from these paired samples were stimulated with CpG in the presence and absence of AlPO_4_-adsorbed TT for 5 days. Stimulation of PBMC from donors before vaccination did not result in the generation of anti-TT IgG-secreting cells (Fig. 1b, c), although release of IgG was intact in controls (Supplementary Fig. 1a). By contrast, anti-TT IgG-secreting cells were detected in the corresponding samples of 8 out of 11 donors post vaccination (Fig. 1b, c; see Supplementary Fig. 1b for total IgG and single donor values). The specificity of the TT response was controlled by stimulation with AlPO_4_-adsorbed diphtheria toxoid (DT). The results confirmed the specificity of TT-specific antibody detection. However, cells from two freshly vaccinated donors with high levels of anti-TT IgG enzymatic reactivity also displayed a positive despite lower response to DT, while all other samples (before and after vaccination) showed a negative TT-specific IgG response (Fig. 1b and Supplementary Fig. 1).

### Frequency of TT-specific B cells and induction of TT-specific IgG-secreting cells in PBMC from buffy coats

Since obtaining the samples from freshly vaccinated donors is logistically challenging, we evaluated the feasibility of use of PBMC from buffy coats. In a preliminary study with 26 donors, we confirmed that TT-specific switched memory IgG-positive B cells are detectable in buffy coat-derived PBMC and are not subject to relevant variance (Supplementary Fig. 2). TT-specific B cells accounted for 0.13 ± SD 0.1521% of IgG+ memory B cells, which corresponds to 0.012% of total peripheral B cells.

We subsequently performed the assay with buffy coat-derived PBMC. Since the TT bulk antigen used in the experiments was adsorbed to alum adjuvant, we included controls with alum adjuvant alone. Thus, PBMC were stimulated with CpG and either TT or DT adsorbed to AlPO_4_ or AlPO_4_ alone (Fig. 1d and Supplementary Fig. 3). The results showed that exposure to alum-adsorbed TT-induced differentiation of anti-TT IgG-secreting B cells. By contrast, aluminum phosphate alone or in combination with DT did not induce B cells producing anti-TT IgG, thus confirming the applicability of buffy coat-derived PBMC and the specificity of the assay.

### Stimulation with TT distinguishes responsive and non-responsive PBMC donors

To study the robustness and reliability of the assay, we carried out experiments with PBMC of 101 buffy coat donors. Initial stimulation was carried out with four technical replicates in each condition. As expected, the results showed significant donor variability. After stimulation with AlPO_4_-adsorbed TT in combination with CpG anti-TT IgG-producing cells were increased in 41 donors when compared to CpG only (Supplementary Fig. 4a). However, 60 out of 101 donor cells were categorized as non-responsive because either enzymatic activity of anti-TT IgG remained below the baseline (i.e., 200), or anti-TT IgG levels after stimulation with CpG only superseded those of TT + CpG.

**Fig. 2:**
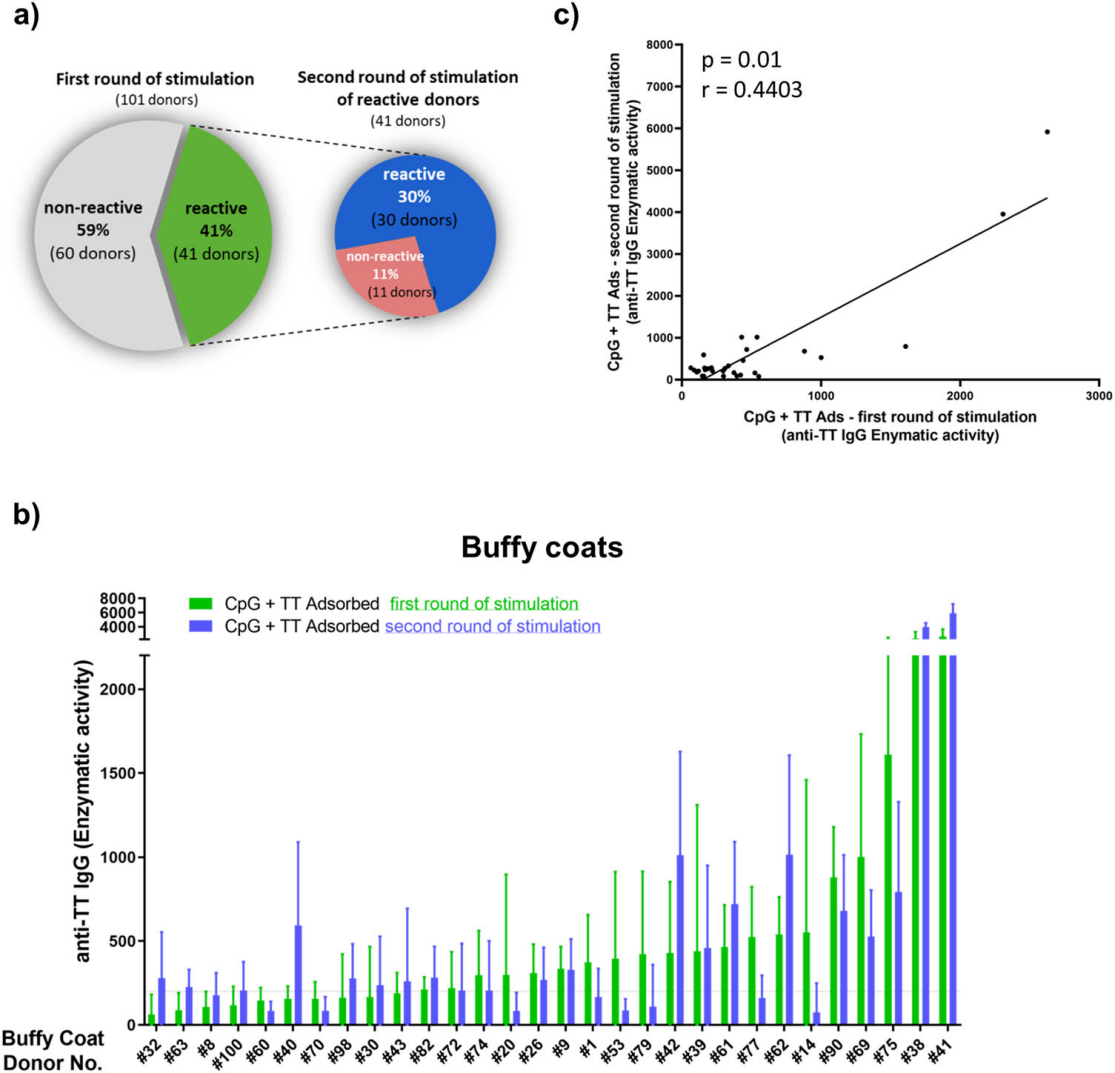
Variability of TT responses among buffy coat donors. **a** Overview of the results from two consecutive rounds of experiments in 101 and 41 donors, respectively. **b** Means of enzymatic activity of anti-TT IgG produced in response to CpG + TT from 30 donors in first and second round of experiment (Donors are arranged according increasing mean value of anti-TT IgG enzymatic activity in a first round of experiments). Bar graphs represent mean (+ standard deviation) of all technical replicates (four and eight technical replicates per donor cell sample in first and second experiment, respectively). First round of experiments: *n* = 101 independent donors and *n* = 11 independent experiments. Second round of experiments: *n* = 41 independent donors and *n* = 9 independent experiments. **c** Spearman correlation of means of enzymatic activity anti-TT IgG response to CpG + TT from first and second round of experiment (30 reactive donors).

To evaluate reproducibility of results, we repeated the experiments with the PBMC from responsive donors. In the second round, under the same conditions, we used eight technical replicates per condition to increase the cell volume tested (Supplementary Fig. 4b). Out of the 41 re-evaluated PBMC samples we confirmed reactivity to AlPO_4_-adsorbed TT in only 30 donors, corresponding to 30% of the *n* = 101 tested buffy coats (Fig. 2a). The mean enzymatic activities obtained with the responsive cells in both the first and second rounds are depicted in Fig. 2b. Compared with high variability among donors, the intra-assay variability at individual donor level was negligible, e.g., enzymatic activity levels display a statistically significant correlation (*p* = 0.01) (Fig. 2c). Slight differences in enzymatic activity levels reflect the expected inter-experimental variation. The high standard deviations on individual donor level depicted in Supplementary Fig. 4 result from variation among the technical replicates, accounting for the differences in the number of TT-specific B cells in each well (e.g., per 2 Mio of PBMC). For future developments, this could be avoided by pooling of technical replicates before analysis. However, the variation shown manifests the need for acquisition of data from high PBMC numbers.

### Switched memory B cells counts correlate with TT responsiveness

**Fig. 3:**
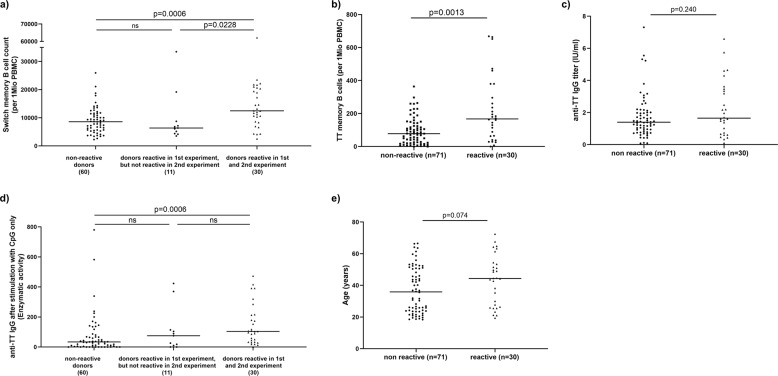
Definition of prequalification criteria for PBMC use. Phenotypical and functional comparison of responsive and non-reactive donors from 101 buffy coat donors. **a** Switched memory B-cell count/10^6^ PBMC, **b** TT-specific switched memory B cells/10^6^ PBMC, **c** levels of anti-TT IgG in plasma, **d** enzymatic activity of anti-TT IgG response to CpG only, and **e** age of non-responders and responsive buffy coat donors. Data were analyzed using Mann–Whitney test. *P* values are depicted in the respective graphs, ns non-significant.

Since prequalification of PBMC might represent a suitable strategy to improve robustness of the assay, we characterized PBMC in regard to B-cell counts and proportional representation of B-cell subpopulations (Table 1) and correlated the findings with reactivity of the PBMC. Analysis of the eleven donors that were reactive only once revealed that these PBMC were characterized by low counts of switched memory B cells (CD19+IgM-CD27+) per 10^6^ PBMC when compared with reactive donors (Fig. 3a), which could explain the variability of the response. Comparison of reactive and non-reactive donors revealed that the switched memory B-cell counts were significantly higher in donors with confirmed reactivity (Fig. 3a).

**Table 1:** Buffy coat donors’ characteristics—data from 101 buffy coats.

Parameter	Median (±SD)	Min—max
Lymphocytes (%)	80 (±6.1)	54–92
B lymphocytes (%)	8 (±3.1)	2–18
Lymphocytes/1 Mio PBMC	800,000 (±60,975)	544,000–921,000
B-cell count/1 Mio PBMC	60,975 (±24,791)	17,250–127,565
Naive B cells (%)	63 (±15.4)	20–91
IgM memory B cells (%)	19 (±11.1)	3–57
Isotype switched memory B cells (%)	16 (±8.9)	9–80
Isotype switched memory B cells count/1 Mio PBMC	9 094 (±7877)	2283–62,157
Anti-TT IgG (IU/ml)	1.5 (±1.5)	0.1–7.3

Since the frequency of TT-specific memory B cells was found to be very consistent in relation to the IgG-positive memory B cells subpopulation (Supplementary Fig. 2), the experimental use of higher total memory B cells increased frequencies of TT-specific switched memory B cells and TT reactivity. This was reflected by a strong correlation (*p* < 0.0001) of TT-specific memory B-cell counts with counts of switched memory B cells per 1 Mio PBMC (Supplementary Fig. 5a). TT-specific memory B-cell counts were further increased in reactive PBMC (Fig. 3b). There was further a positive correlation of initial counts of switched memory B cells (*p* = 0.003) and TT-specific memory B cells (*p* < 0.0001) with the enzymatic activity of anti-TT IgG (Supplementary Fig. 5b, c).

We subsequently analyzed the correlation of anti-TT IgG levels in plasma with functional reactivity in the TT-specific B-cell assay. Despite correlation of anti-TT IgG levels with TT-specific memory B cells (Supplementary Fig. 5d), statistical analysis showed no difference in anti-TT IgG levels from non-reactive and reactive donors (Fig. 3c). Anti-TT IgG in plasma was thus not predictive for reactivity. However, induction of anti-TT-IgG-secreting cells upon polyclonal stimulation of PBMC with B-cell-active TLR9 ligand CpG alone revealed that reactive donors reach significantly higher values (Fig. 3d), which is well in line with a higher number of TT-specific memory B cells (Fig. 3b).

Finally, we asked whether the age of the buffy coat donors would need to be taken into account. Although a trend towards higher age in the responsive donors was observed, no statistical difference in regard to age distribution was found in the responsive and non-responsive groups (Fig. 3e).

### Alum does not alter specificity but influences secretion of TT-specific IgG

**Fig. 4:**
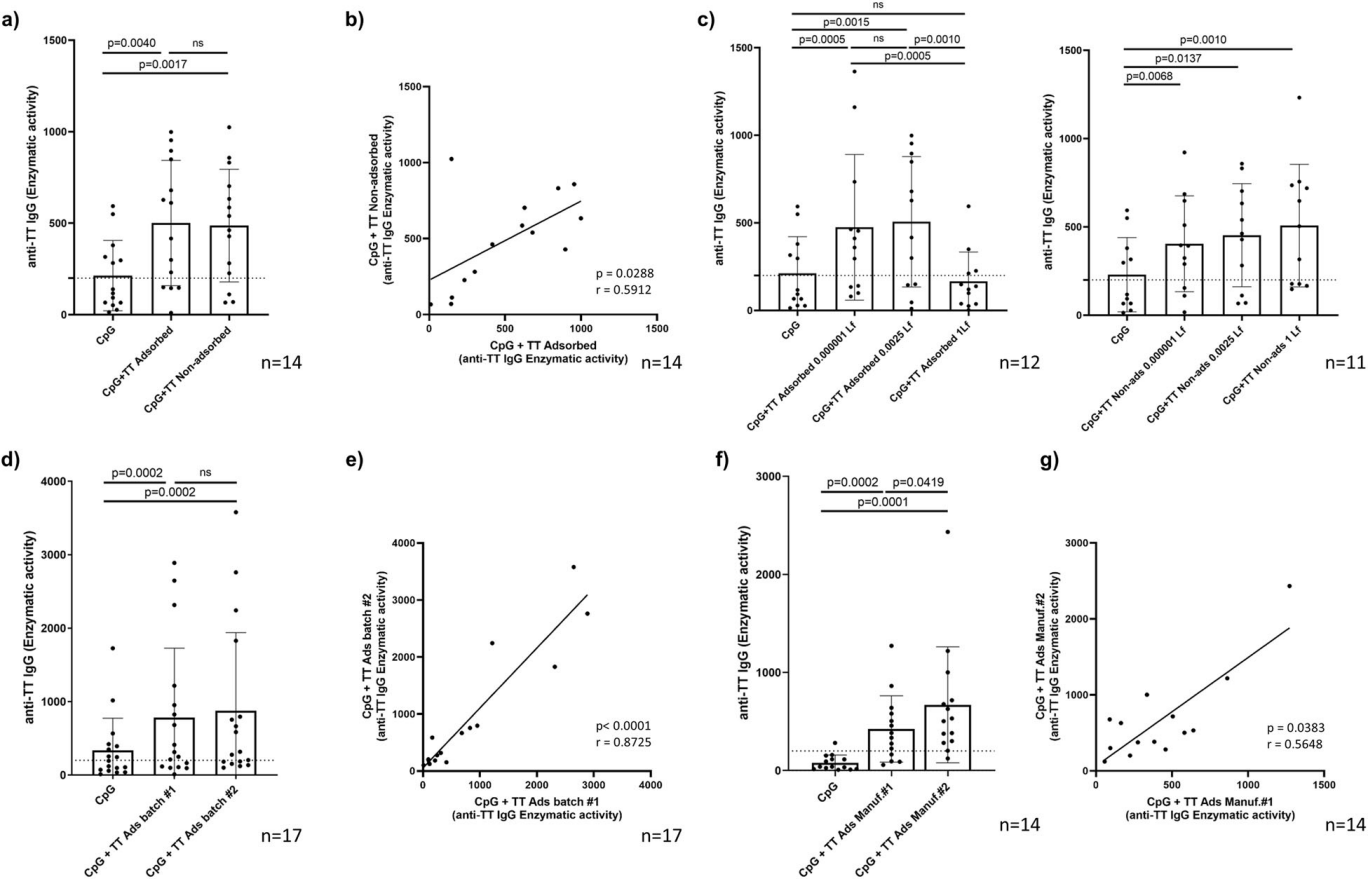
Influence of adjuvant and batch-dependency of the response. Impact of TT adsorption on alum and antigen concentration on anti-TT IgG production in buffy coat donors. **a** Comparison of response to TT adsorbed vs non-adsorbed (0.0025 Lf/ml) (14 donors; *n* = 7 independent experiments), **b** correlation of the enzymatic activity of anti-TT IgG in response to TT adsorbed and non-adsorbed (0.0025 Lf/ml) of individual donors (each represented as a single dot), **c** titration of TT adsorbed (left panel) (12 donors; *n* = 7 independent experiments) and titration of TT non-adsorbed (right panel) (11 donors; *n* = 7 independent experiments). **d** Comparison of the enzymatic activity of anti-TT IgG in response to two different batches from the same manufacturer (17 donors; *n* = 4 independent experiments) and **e** correlation of the means of enzymatic activity of anti-TT IgG of the individual donors in response to the batch #1 and #2 (each donor represented as a single dot) (*n* = 4 independent experiments). **f** Comparison of the enzymatic activity of anti-TT IgG in response to adsorbed TT from two different manufacturers (14 donors; *n* = 3 independent experiments) and **g** correlation of the means of enzymatic activity of anti-TT IgG of the individual donors in response to the TT adsorbed from Manufacturer #1 and #2 (each represented as a single dot). Bar graphs represent mean (± standard deviation) of the enzymatic activity means of all donors. The dotted line shows anti-TT IgG enzymatic activity of 200, the threshold of reactivity. Data were analyzed using Wilcoxon matched-pairs signed rank test and Spearman correlation. *P* values are depicted in the respective graphs, ns non-significant.

Aluminum salts commonly used for TT adsorption such as aluminum hydroxide or phosphate enhance the immunogenicity of the vaccine antigens. We stimulated the cells with CpG in combination with adsorbed and non-adsorbed TT (Fig. 4a). The results showed that production of anti-TT IgG produced was comparable and the presence of aluminum phosphate did not alter the response level, while on individual donor level the means of enzymatic activities in both conditions correlated well (*p* = 0.03) (Fig. 4b).

Since it has repeatedly been claimed that alum is cell-toxic, we asked whether increasing amounts of antigen and adsorbed adjuvant would affect the results. The results showed that enzymatic activities derived from anti-TT IgG-secreting B cells produced in response to 0.000001 Lf/ml or 0.0025 Lf/ml TT were comparable and independent of the adsorption with alum (Fig. 4c). However, 1 Lf/ml of adsorbed TT leads to a statistically significant reduction in enzymatic activity, which was not seen with non-adsorbed TT bulk antigen (Fig. 4c).

### TT batches induce comparable amounts of TT-specific IgG

Since the objective of the study was to develop an assay that could be used for consistency testing in quality control and batch release of bulk antigen and vaccine, we evaluated the induction of TT-specific ASC in response to two different batches of the alum-adsorbed TT bulk antigen from the same manufacturer (Fig. 4d). Levels of anti-TT IgG enzymatic activity were comparable, which was further supported by a very strong correlation (*p* < 0.0001) of anti-TT IgG enzymatic activity on an individual donor level (Fig. 4e).

### Quantitative TT response differs depending on the manufacturer

To further understand the precision of the assay, we studied the anti-TT IgG responses to bulk antigen from different manufacturers. The antigen preparations differed in the adjuvant used for adsorption, e.g., aluminum phosphate versus aluminum hydroxide. The results showed that anti-TT IgG secretion was elicited by both TT preparations, even though the reaction to that provided by manufacturer #2 was consistently higher than that of manufacturer #1 (Fig. 4f). Notably, TT adsorbed from manufacturer #2 contained 10 times higher concentration of aluminum Al^3+^. Importantly, anti-TT IgG responses of adsorbed TT from two manufacturers using different alum adjuvants demonstrate statistically significant correlation on individual donor level (*p* = 0.04) (Fig. 4g).

### Heat alteration of TT does not change levels of anti-TT IgG

**Fig. 5:**
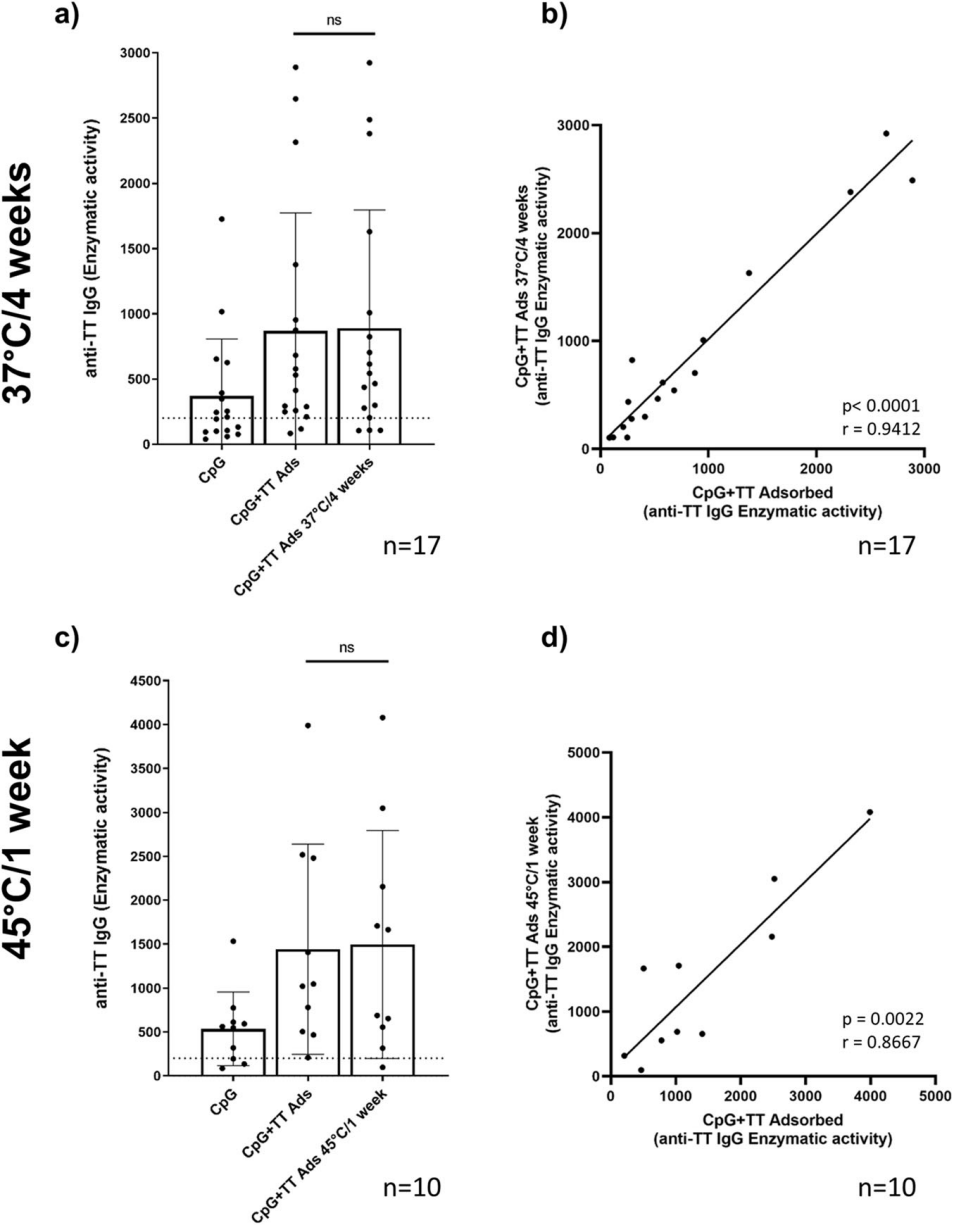
Influence of heat alteration of TT. Anti-TT IgG enzymatic activity of buffy coat donors in response to heat alternated adsorbed TT (0.0025 Lf/ml): **a**, **b** TT kept at 4 weeks at 37 °C (17 donors; *n* = 5 independent experiments) and **c**, **d** TT kept one week at 45 °C (10 donors; *n* = 2 independent experiments). **a**, **c** comparison of response to not-altered vs altered adsorbed TT. **b**, **d** correlation of the enzymatic activity of anti-TT IgG in response to not-altered vs altered adsorbed TT of individual donors (each represented as a single dot). Bar graphs represent mean (± standard deviation) of the enzymatic activity means of all donors. The dotted line shows anti-TT IgG enzymatic activity of 200, the threshold of reactivity. Data were analyzed using Wilcoxon matched-pairs signed rank test and Spearman correlation. *P* values are depicted in the respective graphs, ns non-significant.

Next, we tested whether the assay detects heat alteration of TT. To this end, we used two different heat treatment protocols: TT was either incubated at 37 °C for 4 weeks or at 45 °C for 1 week. The results shown in Fig. 5 and Supplementary Fig. 6 revealed no statistically significant change in enzymatic activity levels. However, heat treatment at 45 °C increased variability of results, which was reflected by a higher standard deviation, an increased *p* value, a decreased correlation coefficient and increased scattering of single values along the trend line when compared with the less-rigorous protocol at 37 °C.

### Induction of TT-specific IgG secretion by treatment with final vaccine product

**Fig. 6:**
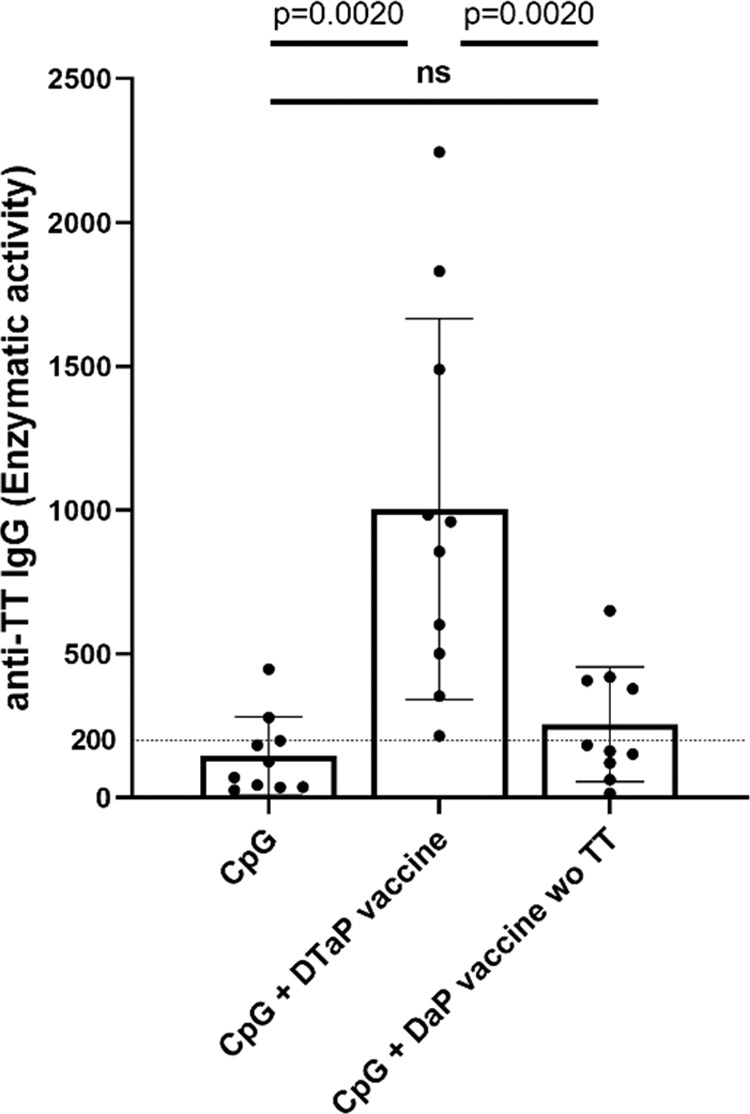
Detection of TT responses with final vaccine product. Anti-TT IgG enzymatic activity in response to CpG + DTaP and DaP vaccine without TT in buffy coat donors. Combined values of enzymatic activity from 10 buffy coats donors with statistical evaluation (Wilcoxon matched-pairs rank test) (*n* = 4 independent experiments). Bar graphs represent mean (± standard deviation) of the enzymatic activity means of all donors. The dotted line shows anti-TT IgG enzymatic activity of 200, the threshold of reactivity. *P* values are depicted in the respective graphs, ns non-significant.

The ultimate goal of this assay development is to test final vaccine product batches. Thus, we tested the response of reactive donor PBMC to final DTaP vaccine product. A drop-out DaP sample lacking TT was used as a control. The results obtained in these experiments showed a TT-specific response that was absent when PBMC were stimulated with the drop-out sample (Fig. 6 and Supplementary Fig. 7). These findings indicated that the assay is compatible with use of final vaccine product.

## Discussion

It is well known that memory B cells enable rapid responses upon re-encounter with a known pathogen^31^. These responses require expansion and subsequent differentiation of antigen-specific B cells into antibody-producing cells. Use of ELISpot is well established for detection of antigen-specific memory B cells and determining the frequency of pathogen and vaccine-specific B cells^25,27,28,32–35^. However, the published protocols focus on the enumeration of antigen-specific B cells and are therefore based on polyclonal stimulation (Bernasconi et al. 2002; Crotty et al. 2004; Weiss et al. 2012; Jahnmatz et al. 2013), which was used for characterization of PBMC in Fig. 3d. By contrast, in this study, we were interested in selective induction of terminal differentiation of antigen-specific B cells to test the quality of the vaccine antigen. Our protocol, thus, builds on the existing pool of TT-specific memory B cells. Although a TLR ligand was used to facilitate proliferation, survival, and differentiation of TT-specific B cells, the low concentration of CpG ODN used avoided unselective expansion and differentiation of B cells in the absence of a BCR stimulus (i.e., TT) (Fig. 1b–d and Supplementary Fig. 1 and 3). This permitted the establishment of an assay that confirms functional integrity.

In this study, assay development was facilitated by the fact that the frequency of TT-specific B cells in peripheral blood is high when compared with other antigens. According to the literature, it can reach up to 0.14% of IgG-producing memory B cells, which approximately represent 0.02% of all circulating B cells^36–39^. This percentage corresponded well with the number of TT-specific IgG-positive B cells we determined in buffy coat-derived PBMC. Using flow cytometric analysis we estimated the frequency of IgG memory B cells at 0.13%, which corresponds to 0.012% of total peripheral blood B cells (Supplementary Fig. 2).

According to the literature, TT-specific plasma cell counts peak around day 7 after booster vaccination^37,40^, whereas the anti-TT memory B-cell count reaches its maximum in peripheral blood 1–2 weeks after immunization^24,37^. Here, proof-of-concept was demonstrated in experiments using paired samples from healthy donors before and after booster DTaP vaccination. The results showed that immunization increased TT-specific IgG-secreting cells (Fig. 1b). Notably, the response to TT was not detectable before booster immunization. In light of previous vaccination dating back between 5 and 26 years, this was well in line with the gradual drop in memory B-cell counts in peripheral blood described subsequent to vaccination^24,37^ and highlighted the requirement for booster immunization to pass the detection threshold of the assay. However, similarly to the results obtained in buffy coat-derived PBMC where 30% of donors were reproducibly responsive, some donors (# 4, 6, and 8; e.g., 3 of 11 donors) were unresponsive. The reasons for this finding remain to be clarified: no difference was identified based on years elapsed from previous vaccination, number of days post vaccination, switched memory B-cell counts or levels of anti-TT antibodies in plasma. Furthermore, we can only speculate whether unspecific responses to DT reflect elevated activation of antibody-secreting cells after vaccination.

Since the assay builds on a secondary immune response, it was further not surprising that PBMC from buffy coats displayed a broadly variable range of reactivity (Supplementary Fig. 4). Nevertheless, in this study, we took advantage of the high coverage with TT vaccination in the German population. Here, ~70–75% of adults were vaccinated in the last 10 years^3,7^. Although we presumed that blood donors were likely to have received TT vaccination in the past, information on the individual history of vaccination and the time elapsed from the last (re)vaccination was not available. We reduced the impact of this variability on the performance of our assay by two measures: (i) to increase sensitivity we performed experiments using a high number of technical replicates with the aim of increasing the total amount of B cells analyzed per sample (Fig. 1b, Supplementary Fig. 1, Supplementary Fig. 3), thus accounting for samples with low frequency of TT-specific memory B cells; (ii) we further sought to identify criteria predictive of donor reactivity and subsequently developed prequalification protocols to enable reliable routine use of cryopreserved PBMC.

**Fig. 7:**
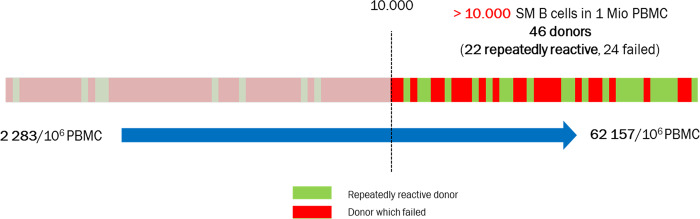
Threshold for reactivity of donors. A threshold of >10,000 memory B cells per 10^6^ PBMC can increase the proportion of reactive donors. This criterion would have reduced the number of donors to be subjected to functional evaluation to 46 of 101 donors; 22 (48%) of these PBMC samples would have been repeatedly reactive and the proportion of non-responders in pre-testing could have been reduced from 70 to 52%.

When we studied the relationship between donor reactivity and memory B-cell counts, we observed that reactive donor cells on average contained higher counts of switched memory B cells than donors whose cells were not reactive or with reactivity not reproducible upon repetition (Fig. 3a). This observation implied that a higher proportion of memory B cells also results in higher numbers of TT-specific memory B cells. Taking into account that the use of technical replicates increased sensitivity of the assay, we concluded that a minimal representation of memory B cells could serve as a prequalification criterion for inclusion of donor PBMC into the biobank. Indeed, a threshold of >10.000 memory B cells per 10^6^ PBMC would have increased the proportion of reactive donors: 22 of 46 (48%) PBMC samples fulfilling this criterion would have been repeatedly reactive (Fig. 7). Most importantly, all donor cells reactive in the first but not in the second round of stimulation would have been excluded by application of this criterion.

Precise assessment of the number of TT-specific memory B cells could represent an alternative parameter for prequalification. The enumeration of anti-TT IgG-secreting cells following polyclonal B-cell stimulation was discarded because it provided no advantage compared with flow cytometric quantification of switched memory B cells and requires more time and cost. Notably, this criterion did not reliably distinguish reactive from non-reactive donors (Fig. 3b) we favored total switched memory B-cell counts. Furthermore, direct quantification of this rare subpopulation by flow cytometry requires extensive event collection, ideally performed with enriched B cells, which would be accompanied by significant cell loss and was therefore, also abandoned.

Assuming that anti-TT IgG levels might correlate with PBMC reactivity, we quantified anti-TT IgG in plasma obtained from buffy coats (Fig. 3c). In agreement with previous reports, the numbers of TT-specific memory B cells did not correlate with anti-TT IgG levels^36,41^ nor was there any statistically significant difference between non-responders and reactive donor cells.

Since BCR affinity to antigen increases with the number of somatic hypermutations introduced by repeated encounter of the antigen^42–44^, we hypothesized that affinity maturation of the TT-specific BCR repertoire, in particular avidity to TT, could account for the missing correlation of TT-specific memory B-cell numbers with reactivity. This premise was well in line with a trend towards a higher age in reactive donors when compared with non-responders (Fig. 3e). However, follow-up studies will need to explore the relevance and the feasibility for preselection of reactive donors. Based on the observations made, we propose a two-step approach for prequalification of donor cells: (1) initial selection of PBMC based on memory B-cell counts can avoid unnecessary testing of non-reactive donors; and (2) the remaining samples need to be functionally tested for reactivity to TT stimulation. In addition, we propose that the donor cell qualification criteria could be amended by a minimal requirement for reactivity in two out of six wells. Optionally, the assay performance could be improved by increasing the number of technical replicates (e.g., 12). This approach represents an important means for reduction of variability, standardization and insurance of robustness, and reproducibility of the B-cell assay.

Since the objective of our study was to develop an assay that would be suitable for vaccine antigen characterization and quality control of TT vaccines, we explored the limits of the methodological approach with a variety of different materials provided by vaccine manufacturers. The results confirmed that the assay is specific for TT in a wide range of the antigen concentrations (Fig. 4c) (0.000001 Lf/ml to 0.0025 Lf/ml) with no interference attributable to the presence of alum adjuvant in absorbed TT up to 0.0025 Lf/ml (Fig. 4a, b). Furthermore, no toxic effect was observed when final vaccine product was analyzed (Fig. 6). Since batches of bulk antigen are produced at high-quality standards, it is not surprising that the anti-TT IgG responses of two different batches were comparable and showed tight correlation on individual donor level (Fig. 4d, e), thus demonstrating consistency in the immune stimulatory effect.

Interestingly, we observed differences in the strength of response when PBMC were stimulated with TT from different manufacturers (Fig. 4f) although TT bulks might be expected to contain identical epitopes. This finding might, thus, be attributable to the different adjuvants used for TT adsorption (e.g., aluminum phosphate versus aluminum hydroxide) or the respective amount of adjuvant. Thus, the assay detects differences in adjuvant and its complexion with the antigen. However, it cannot be excluded that the results were influenced by the donor history of vaccine products used for previous booster vaccinations. Nevertheless, anti-TT IgG levels obtained from individual donor cells correlated well (Fig. 4g), reflecting consistency and suitability for comparative analysis of antigens.

Notably, the experiments with heat-treated TT samples did not show statistically significant differences compared with the non-altered TT (Fig. 5). However, we have no evidence that heat exposure induced alterations affecting the B-cell epitopes. Thus, the current assay design allows conclusions on the immunogenicity of TT antigen but cannot distinguish alternated TT if immunodominant epitopes are preserved.

Even though vaccines containing TT have been used for >90 years^45^ and are produced under GMP conditions, ensuring consistent quality of TT vaccines requires vaccine batches to be tested before release. To date, these tests include in vivo potency assays performed as either challenge or immunogenicity studies. Altogether, the method evaluated in this study provides an easy in vitro tool to monitor presence and immunogenic potential of vaccine antigen based on eliciting a recall response from preformed antigen-specific memory B cells. Our results demonstrate the feasibility and specificity of an in vitro assay for functional testing of TT in bulk antigen and final vaccine product. Importantly, in contrast to current animal testing, this assay assesses antigen immunogenicity in the context of the human immune response. Furthermore, this response was independent of the presence of adjuvants, other antigens or excipients contained in the final vaccine formulation (Fig. 6). Although the assay in its current form is not suitable for quantification of TT antigen content, functional TT integrity is relevant and can complement biochemical assays in replacing in vivo potency testing. Prequalification of donor cells represents an important asset for reproducibility and reliability of the assay. Follow-up studies focused on reproducibility or sensitivity of the assay in the presence of TT antigen with altered epitopes are needed to further characterize the assay and its limitations. Final method validation further requires a parallel study with the established methodology.

## Methods

### Cell isolation and cryopreservation

Peripheral blood mononuclear cells (PBMC) were isolated from buffy coats from healthy donors obtained from German Red Cross South transfusion center (Frankfurt am Main, Germany) or from healthy volunteers before and 7–26 days after booster DTaP vaccination, which occurred 5–16 years after their previous vaccination. Table 2 summarizes the donor characteristics. The use of buffy coats and the blood collection from healthy donors before and after vaccination were approved by the ethics committee of the University of Frankfurt, approvals #154/15 and #58/16, respectively. Blood of fresh blood donors’ was collected after informed consent was signed. All subjects gave their informed consent for inclusion before they participated in the study and the study was conducted in accordance with the Declaration of Helsinki.

**Table 2:** Vaccinated donors’ characteristics.

Number	Sex	Age	Time since last TT vaccination (years)	Blood taken after vaccination (days)	Anti-TT IgG titer in plasma before vaccination (IU/ml)	Anti-TT IgG titer in plasma after vaccination (IU/ml)	Switched memory B cells in 1 Mio PBMC before vaccination	Switched memory B cells in 1 Mio PBMC after vaccination
Donor #1	M	26	13	26	1,3	9,9	10,015	10,323
Donor #2	F	34	9	26	0,6	12,0	12,928	16,037
Donor #3	M	34	23	15	0,7	1,5	16,639	21,350
Donor #4	M	47	9	18	1,2	3,9	7798	11,684
Donor #5	M	33	6	15	1,4	3,6	17,079	18,386
Donor #6	F	55	7	18	6,6	7,1	7914	7242
Donor #7	M	47	5	20	1,2	3,9	13,996	13,577
Donor #8	F	26	8	14	0,9	9,9	13,834	12,377
Donor #9	F	25	9	20	n.a.	8,4	n.a.	11,945
Donor #10	F	25	8	13	1,2	10,5	12,828	10,895
Donor #11	M	58	10	7	0,6	n.a.	9749	7289

PBMC from donors #4, #6, and #8 after vaccination did not respond to CpG + TT in the assay.

PBMC were isolated by Pancoll gradient centrifugation (PAN-Biotech, Germany). Before cryopreservation, isolated PBMC were centrifuged and resuspended in fetal calf serum (FCS) to the concentration 30 Mio/ml. Aliquots à 15Mio (500 µl of the cell suspension) per freezing vial (CryovialsCryo.S; Greiner Bio-One, Austria) from each donor were prepared. Shortly before vial would be closed, mixed by inverting and placed into slowly cooling container (CoolCell®LX, Biocision, USA) additional 500 µl of freezing media (20% FCS (Sigma-Aldrich Chemie GmbH, Germany), 60% RPMI (RPMI 1640; Gibco, Germany) and 20% DMSO (Dimethyl sulfoxide; Sigma-Aldrich Chemie GmbH, Germany)) were added to the cell aliquot in each vial. Freezing container with vials was immediately placed into −80°C freezer.

### Thawing procedure

Required number of frozen vials of each donor was processed proportionally to the number of vials according to the following protocol. One vial containing 1 ml of cell suspension was placed into 37 °C water bath for 1 min. The cell suspension was transferred into 1 ml of prewarmed (37 °C) thawing medium prepared as 50% FCS, 50% HEK medium (90% RPMI medium, 10% FCS, 1% l-glutamine (200 mM) (Biochrom, Germany), 1% penicillin/streptomycin (10,000 U/ml) (Merck, Germany)) and 180 U/ml of DNAse I (Roche, Switzerland). Followed by immediate addition of 1 ml prewarmed (37 °C) HEK media. Cell suspension was placed for 1 hour in and 37 °C incubator. Afterwards, the cell suspension was centrifuged (360 × *g*, 6 min, RT) and the supernatant was discarded. Cells were resuspended in 1 ml HEK media and counted using trypan blue (ApplichemPanreac, Germany) and Neubauer chamber.

### Cell culture

PBMC were seeded into 48-well plate (Greiner Bio-One, Germany) 2 × 10^6^ per well. Cells in all conditions were stimulated with full-length PTO-modified CpG 2006 (5′-tcgtcgttttgtcgttttgtcgtt-3′) (EurofinsGenomics, Germany) at a final concentration of 0.002 µM. TT adsorbed to alum adjuvant from manufacturer #1 (lot# A and B) and manufacturer #2 (lot# C) were used at a concentration of 0.0025 Lf/ml, if not stated otherwise. Alum-adsorbed DT bulk antigen provided by manufacturer #1 was used at a final concentration of 0.0025 Lf/ml (final concentration of AlPO_4_ 0.052 µg/ml, corresponding to aluminum Al^3+^ concentration 0.011 µg/ml). AlPO_4_ obtained from manufacturer #1 was applied as control at a final concentration of 0.058 µg/ml AlPO_4_ (corresponding to aluminum Al^3+^ concentration 0.013 µg/ml), which is equivalent to the amount in Alum-adsorbed TT conditions (0.0025 Lf/ml). In the experiment, where TT adsorbed from two manufacturers was compared, TT adsorbed from manufacturer #2 was used at a concentration of 0.0025 Lf/ml, the adsorbent Al(OH)_3_ in final concentration of 0.26 µg/ml (corresponding to aluminum Al^3+^ concentration 0.13 µg/ml). DTaP and a DaP drop-out sample lacking TT were provided by manufacturer #1 and used for stimulation at a final concentration of 0.0025 Lf/ml of TT (4000-fold dilution). Wherever possible, each condition was performed in six or eight technical replicates. The final volume of the stimulated cell suspension was 1 ml per well in a 48-well plate. Cells were subsequently incubated in 37 °C, 5% CO_2_ for 5 days.

### ELISpot

MultiScreen HTS IP Durapore PVDF ELISpot plates (Merck, Germany) were coated with 10 Lf/ml TT (AJ vaccines, Denmark) or with 6.25 ng/ml anti-human IgG (clone MT91/145; Mabtech, Sweeden) in PBS overnight at 4 °C. Before cell seeding, the ELISpot plates were washed three times with PBS and block with HEK media for at least 30 min at the room temperature (RT). Cells after 5 days of incubation in 48-well plate were centrifuged (360 × *g*, 6 min, 20 °C) and cell were washed carefully twice with 1 ml of HEK media. After the supernatants were discarded, the cells in each well were resuspended in 400 µl of HEK media. In all, 200 µl of the cell suspension of single well was transferred in single TT coated ELISpot well. For detection of total IgG secretion—190 µl from the other portion of stimulated PBMC was discarded and 400 µl of HEK media was added. From this 40 times diluted cell suspension (to prevent too high number of total IgG spots per ELISpot well), 200 µl of cells were seeded on anti-human IgG coated ELISpot plate. Cells were placed in 37 °C incubator with 5% CO_2_ for 4 hours. The cells were subsequently discarded and plate was washed three times with PBS. Biotinylated anti-IgG detection mAbs MT78/145 clone (Mabtech, Sweden) at a concentration of 0.001 mg/ml in PBS supplemented with 10% FCS were added to the wells and incubated for 2 hours at RT. The plates were washed three times with PBS with 0.05% Tween 20 (Sigma-Aldrich, Germany) before streptavidin conjugated with Alkaline-phosphatase (Becton Dickinson, USA) diluted 1:1000 in PBS with 10% FCS was added and incubated for 1 hour at RT. Plates were washed three times with PBS with 0.05% Tween 20 and three times with PBS. AP-conjugate Substrate kit (BioRad, Germany) was added to the wells for 2–5 min. The reaction was stopped by rinsing the plates with tap water. The plates were dried overnight in dark. Evaluation of plates was done with iSpot ELISpot reader (AID, Germany) with EliSpot Reader v.7.0 (AID, Germany) within 7 days from development.

Production of antibodies is measured as enzymatic activity, which facilitates quantification and directly correlates with the number, intensity and size of the spots within the well. A threshold enzymatic activity of ≥200 was setup as a limit of “reactivity” of the PBMC in a well. This threshold represents minimal limit of eye-visible reaction in a well. Enzymatic activity of 200 is represented in the graphs with a dotted line where applicable.

“Reactive donor cells” were defined by the following criteria: (1) if an enzymatic activity was ≥200 in at least one of six replicate wells stimulated with CpG and TT and (2) if the highest value of the enzymatic activity from the wells treated with CpG and TT was higher than that obtained in all wells that were stimulated with CpG only.

### Enumeration of TT-specific memory B cells by ELISpot

PBMC were plated in 48-well plate at a concentration of 2 × 10^6^ cells per well in 1 ml of HEK media supplemented with R848 (1 µg/ml) (Invivogen, USA) and 10 ng/ml IL-2 (Miltenyi, Germany) and cultured in 37 °C with 5% CO_2_. On day 5 PBMC were washed and seeded in twofold serial dilutions in duplicates (0.5, 0.25, and 0.125 × 10^6^) and incubated for 4 hours on ELISpot plates coated with TT (AJ vaccines, Denmark)^38^. The coating and developing procedures were performed as described above. Frequency of TT-specific memory B cells was determined per 1 × 10^6^ of PBMC.

### FACS staining

For B-cell count and B-cell subpopulations determination, 200,000 PBMC were transferred into 96 U-well plate (Greiner Bio-One, Germany). After PBS was added up to volume of 200 µl, the plate was centrifuged (360 × *g*, 6 min, RT). Cells were stained with 50 µl of mixture of anti-human CD19-Pc7 (clone J3-119; Beckman Coulter, USA), anti-human CD27-BV421 (clone M-T271; Becton Dickinson, USA), anti-human IgM-PerCP Cy5.5 (clone MHM-88; BioLegend, USA) and PBS with 0.5% FCS for 30 min, 4 °C, dark. Afterwards 150 µl PBS with 0.5% FCS was added and the samples were centrifuged (360;× *g*, 6 min, RT), supernatant discarded. The cells were resuspend in 100 µl of PBS, followed by adding 100 μl of 4% PFA (paraformaldehyde) (Sigma-Aldrich Chemie GmbH, Germany) and the suspension was well mixed. The samples were kept in fridge (4 °C) overnight. Next day, the samples were washed before the measurement and cells were resuspended in PBS with 0.5% FCS. Samples were measured on a BD LSRII SORP flow cytometer with BD FACS Diva software version 8.0.1 (Becton Dickinson, USA) and analyzed with Kaluza Analysis Software (Beckman Coulter, USA). The gating strategy is shown in Supplementary Fig. 8.

### Flow cytometric enumeration of TT-specific memory B cells

Before staining, untouched B cells were enriched from PBMC using the EasySep human B-cell enrichment kit (StemCell Technologies, Canada). TT-specific B cells in buffy coat PBMC were then characterized with an antibody panel consisting of anti-human CD19-Pc7 (clone J3-119; Beckman Coulter, USA), anti-human CD27-BV711 (clone O323; BioLegend, USA), anti-human IgM-BV605 (clone MHM-88; BioLegend, USA), anti-human IgA (clone REA1014; Miltenyi, Germany) and with TT purchased from AJ vaccines, Denmark, biotinylated with EZ-Link Sulfo-NHS-LC-Biotin (Thermo Fisher Scientific, UK) and fluorescently labeled with streptavidin-BV421 (BioLegend, USA). Samples were measured on a FACS Aria™ Fusion (Beckman Coulter, USA). The gating strategy is depicted in Supplementary Fig. 2c.

### ELISA

Levels of anti-TT IgG antibodies in plasma of were evaluated by human anti-TT IgG EIA Kit (The Binding Site, United Kingdom) according to the protocol provided by the manufacturer.

### Statistical analysis

Statistical analysis of results was performed using GraphPad Prism 8.4.2 (GraphPad Software LLC, USA). Data were analyzed using two-tailed Wilcoxon matched-pairs signed rank test, two-tailed Mann–Whitney test and Spearman correlation. *P* values are depicted in the respective graphs, ns non-significant.

### Reporting summary

Further information on research design is available in the Nature Research Reporting Summary linked to this article.

## Supplementary information

Supplementary Information

Reporting Summary

## Data Availability

All data from this study are available from the corresponding author upon reasonable request.
