# Supplementary material for: A cell-based in vitro assay for testing of immunological integrity of Tetanus toxoid vaccine antigen
Source: NPJ Vaccines. 2021 Jun 23;6:88. doi: 10.1038/s41541-021-00344-1 (PMC8222253; doi:10.1038/s41541-021-00344-1)
Supplement: Supplementary file 1 — Supplementary Information [file 41541_2021_344_MOESM1_ESM.pdf]

Supplementary Figure 1

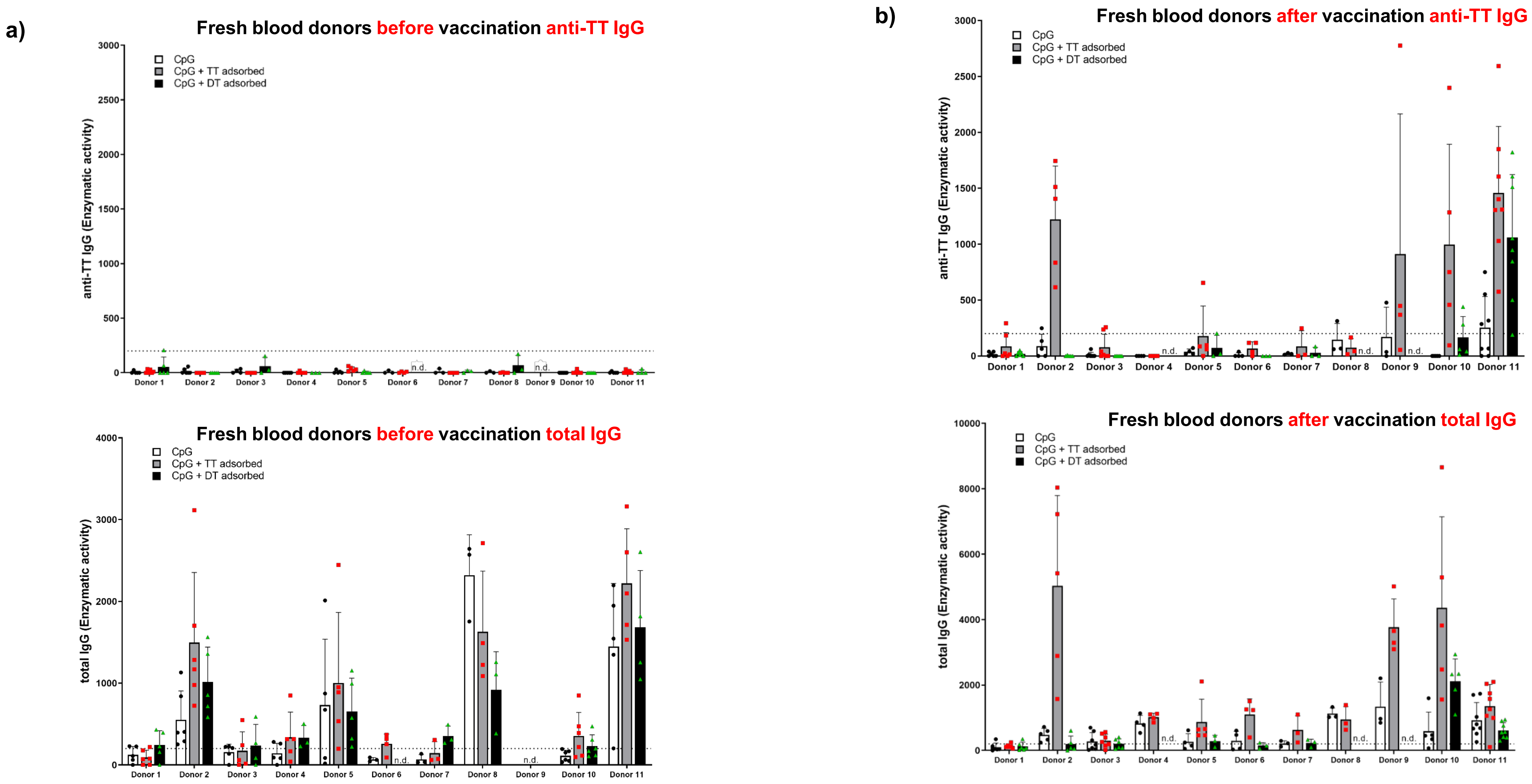

Supplementary Figure 2: a) Anti-TT IgG specific (upper panel) and total IgG (lower panel) enzymatic activity response to the TT adsorbed in assay performed with PBMC obtained from 12 donors **before** and **b) after** DTaP booster vaccination. Bar graphs represent mean (+ standard deviation) of all technical replicates represented as dots (up to six technical replicates per donor cell sample per assay condition) (n=3 independent experiments). The dotted line represents anti-TT IgG enzymatic activity of 200, the threshold of reactivity.

Supplementary Figure 2

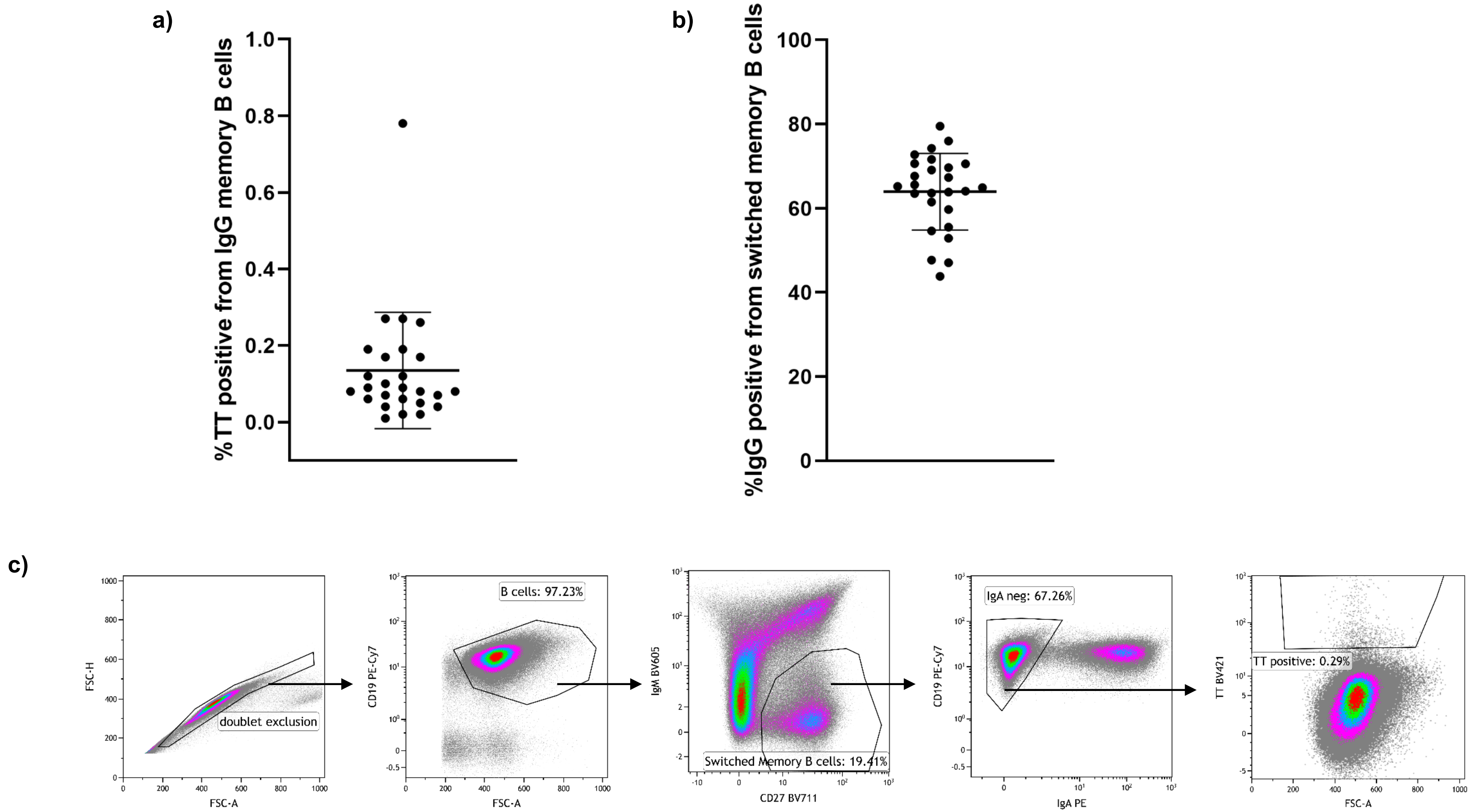

Supplementary Figure 3: a) Frequency of TT positive B cells from IgG memory B cells in buffy coat donors (n=26). **b)** Proportion of IgG positive B cells from switched memory B cell (CD19+CD27+IgM-) (n=26 independent donors and n=12 independent experiments). **c)** Flow cytometry gating strategy for TT-positive IgG+ switched memory B cells.

Supplementary Figure 3

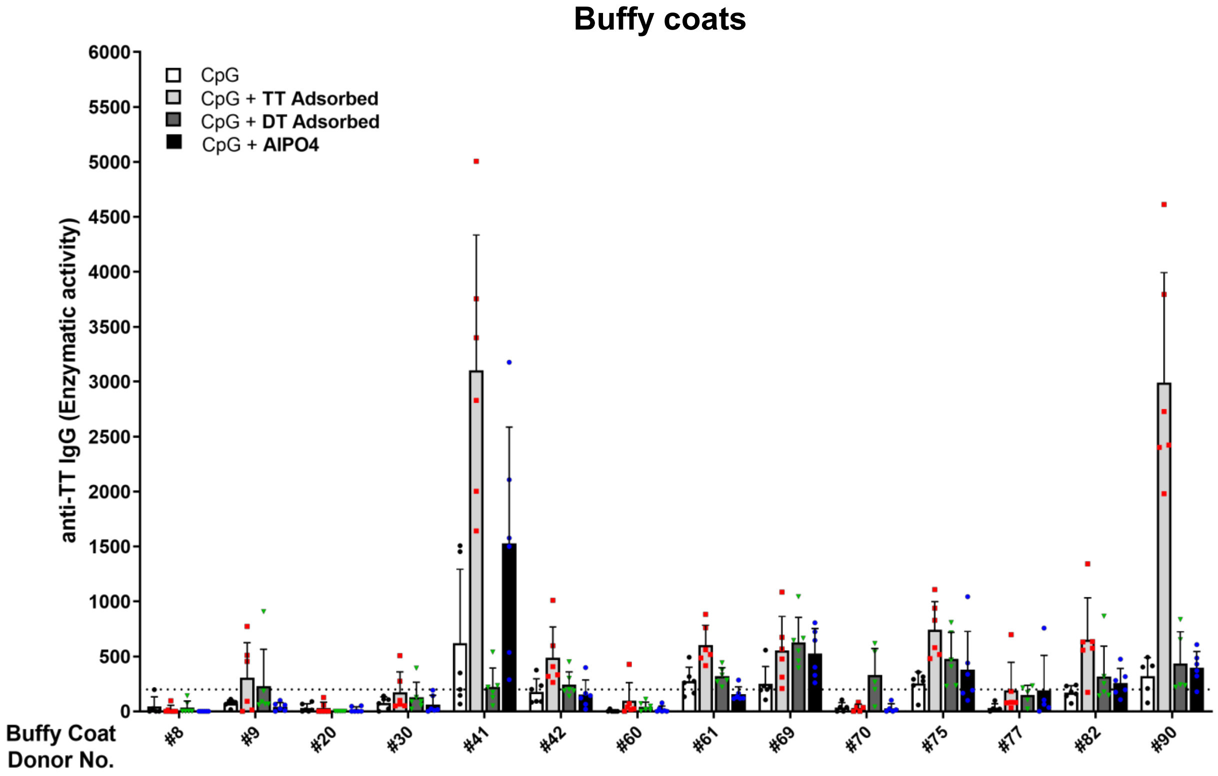

Supplementary Figure 4: Anti-TT IgG enzymatic activity of 14 buffy coat donors in response to CpG + adsorbed TT compared to the response to CpG + adsorbed DT or to CpG + aluminium adjuvant. Single donors' data. Bar graphs represent mean (+ standard deviation) of all technical replicates (five to six technical replicates per donor cell sample per assay condition are represented with the symbols; n=4 independent experiments). The dotted line shows anti-TT IgG enzymatic activity of 200, the threshold of reactivity.

Supplementary Figure 4

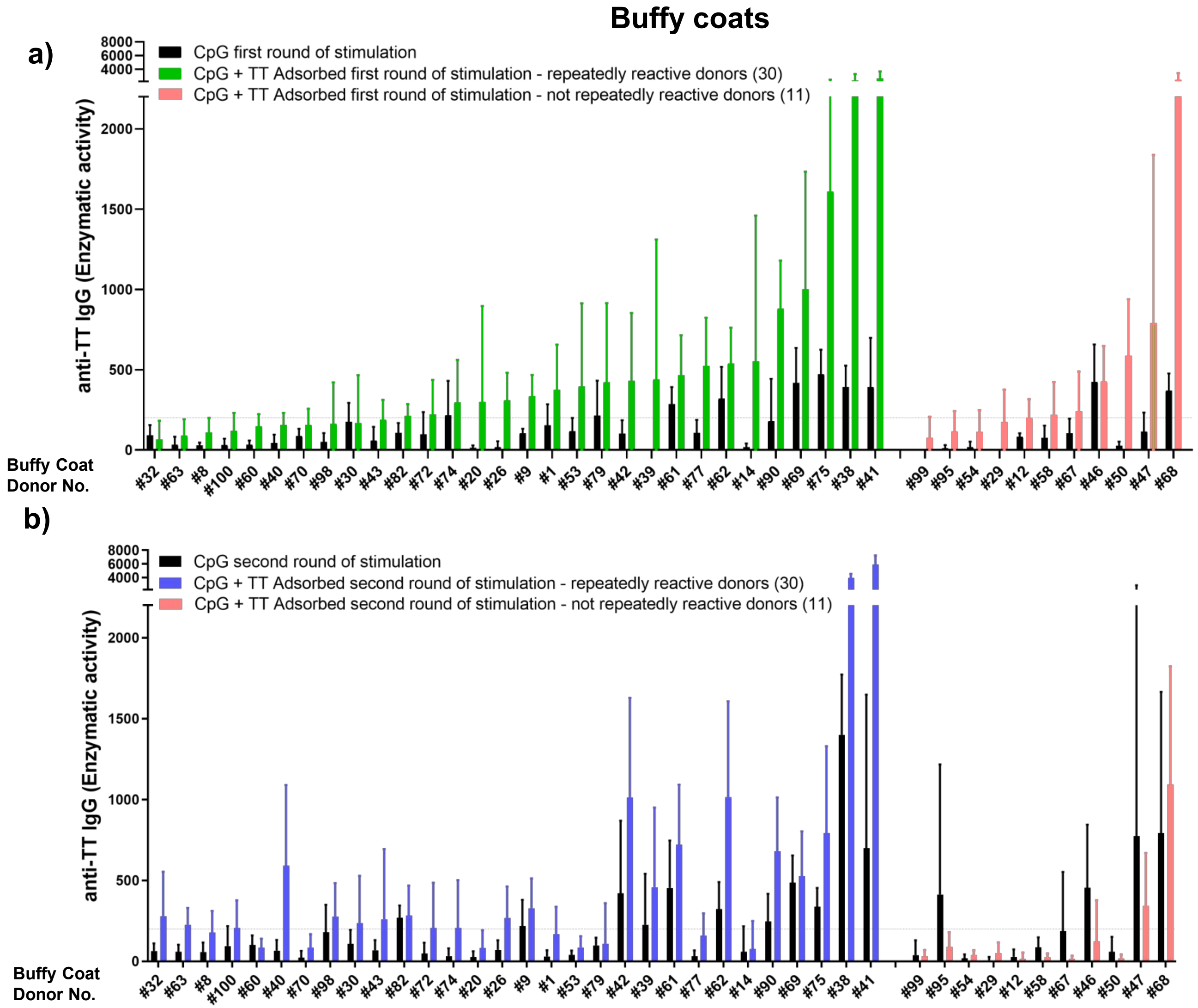

Supplementary Figure 5: Buffy coat donors. **a)** 41 from 101 buffy coat donors showed anti-TT IgG specific enzymatic activity response to TT adsorbed in first experiment. **b)** From 41 reactive donors 30 showed anti-TT IgG specific enzymatic activity response to the TT adsorbed also in the second experiment. Bar graphs represent mean (+ standard deviation) of all technical replicates (in first experiment 4, in second 8 technical replicates per donor cell sample per assay condition). Red bars illustrate values of donors which were reactive in the first experiment, but not reactive in the second experiment. The dotted line shows anti-TT IgG enzymatic activity of 200, the threshold of reactivity. Donors are arranged according increasing mean value of anti-TT IgG enzymatic activity in a first round of experiments. First round of experiments: n=101 independent donors and n=11 independent experiments. Second round of experiments: n=41 independent donors and n=9 independent experiments.

Supplementary Figure 5

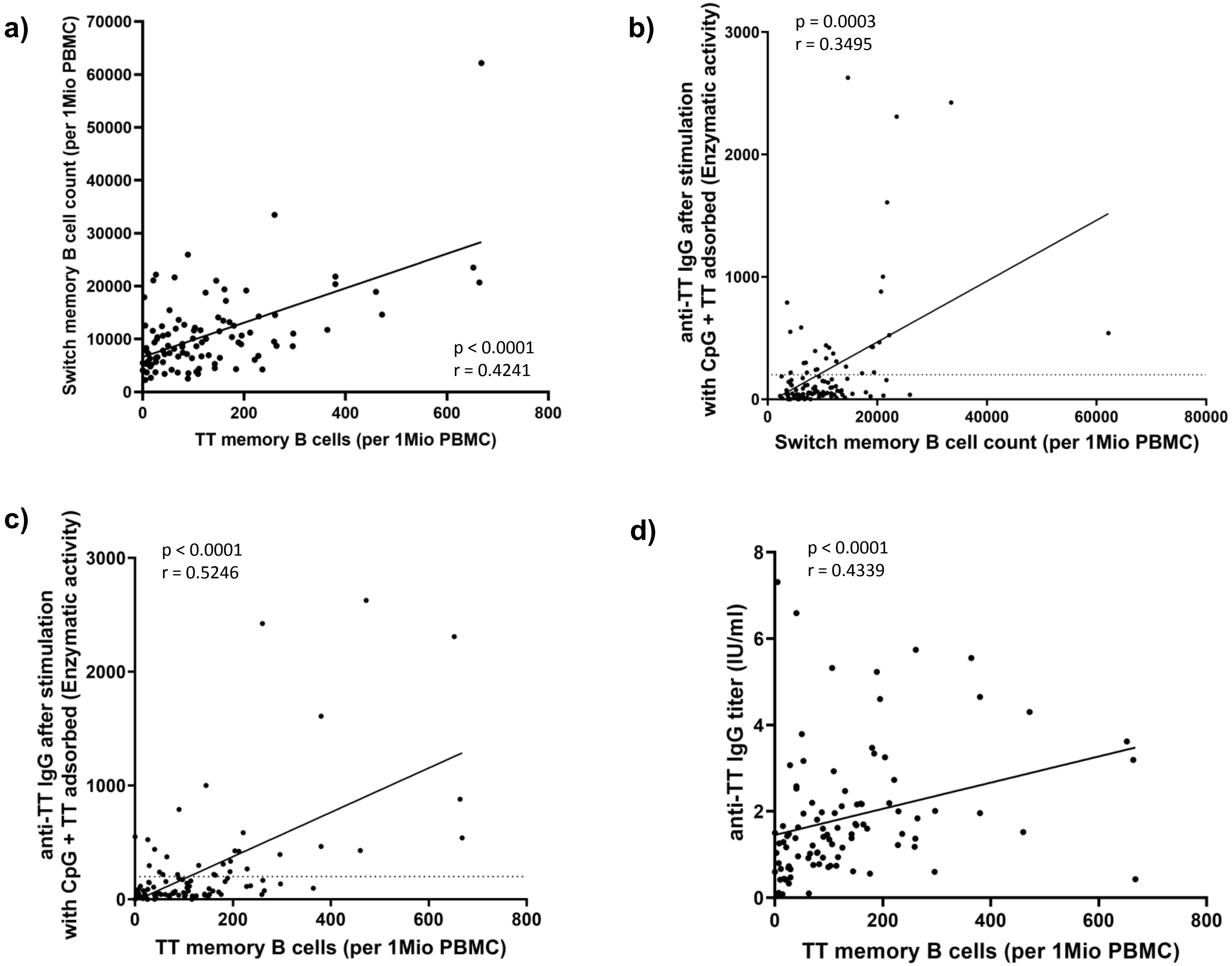

Supplementary Figure 6: Correlation a) of switched memory B cell count per 1Mio PBMC and TT specific memory B cells count, **b)** of switched memory B cell count per 1Mio PBMC and enzymatic activity of anti-TT IgG after the PBMC were stimulated with CpG and TT adsorbed, **c)** of TT specific memory B cells count and enzymatic activity of anti-TT IgG after the PBMC were stimulated with CpG and TT adsorbed and **d)** of TT specific memory B cells count and levels of anti-TT IgG in plasma (data from 101 buffy coat donors).

Supplementary Figure 6

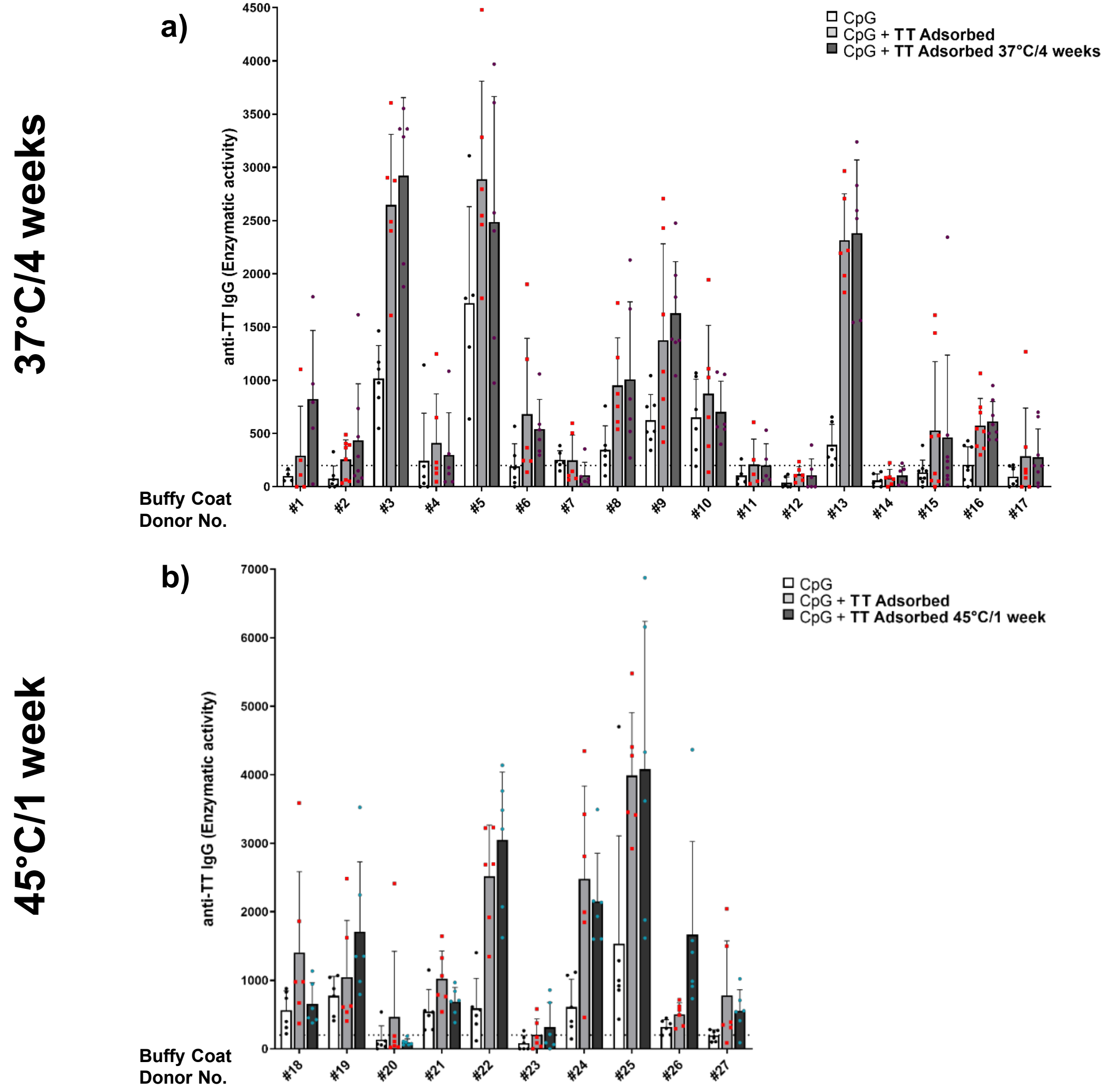

Supplementary Figure 7: Influence of heat alteration of the antigen. Anti-TT IgG enzymatic activity of buffy coat donors in response to heat alternated adsorbed TT (0.0025 Lf/ml). Single donors' data. Bar graphs represent mean (+ standard deviation) of all technical replicates (five to six technical replicates per donor cell sample per assay condition are represented with the symbols). **a) TT kept at 4 weeks at 37°C** (n=5 independent experiments) and **b) TT kept one week at 45°C** (n=2 independent experiments). The dotted line shows anti-TT IgG enzymatic activity of 200, the threshold of reactivity.

Supplementary Figure 7

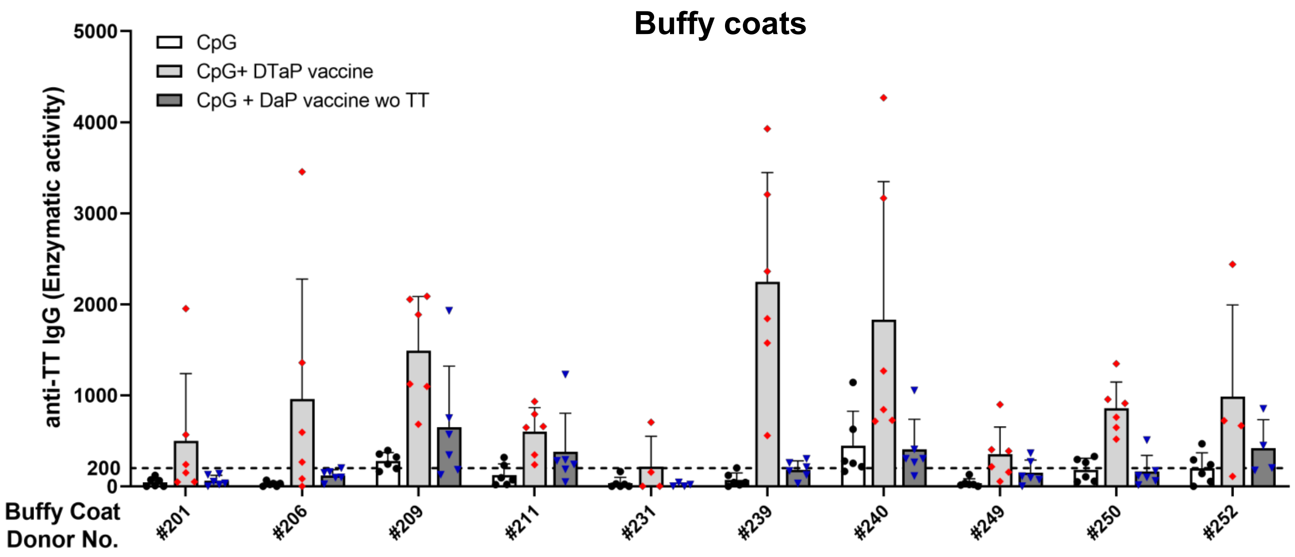

Supplementary Figure 8: Detection of TT responses with final vaccine product. Anti-TT IgG enzymatic activity in response to CpG + DTaP and DaP vaccine without TT. Single donors' data. Bar graphs represent mean (+ standard deviation) of all technical replicates (five to six technical replicates per donor cell sample per assay condition are represented with the symbols; n=4 independent experiments). The dotted line shows anti-TT IgG enzymatic activity of 200, the threshold of reactivity.

Supplementary Figure 8

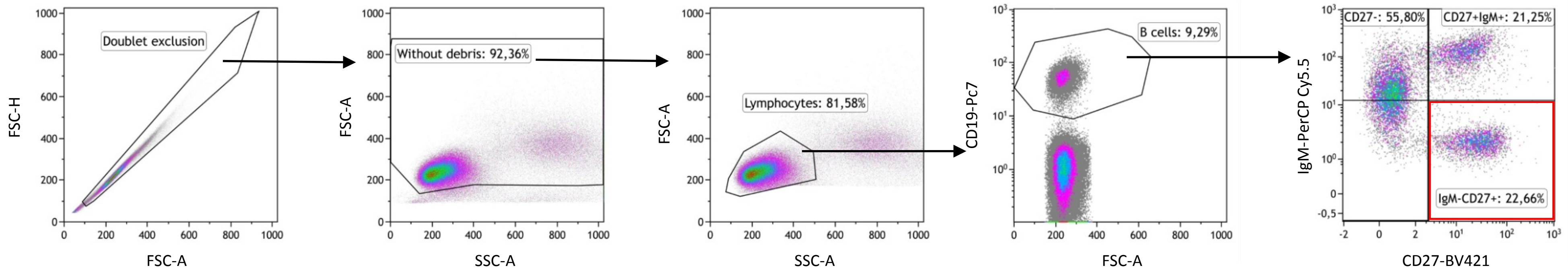

Supplementary Figure 1: Gating strategy for evaluating switched memory B cells percentage by flow cytometry.
