# Supplementary material for: A cell-based in vitro assay for testing of immunological integrity of Tetanus toxoid vaccine antigen
Source: NPJ Vaccines. 2021 Jun 23;6:88. doi: 10.1038/s41541-021-00344-1 (PMC8222253; doi:10.1038/s41541-021-00344-1)
Supplement: Supplementary file 2 — Reporting Summary [file 41541_2021_344_MOESM2_ESM.pdf]

nature research

Corresponding author(s): Isabelle Bekeredjian-Ding

Last updated by author(s): Mar 23, 2021

Reporting Summary

Nature Research wishes to improve the reproducibility of the work that we publish. This form provides structure for consistency and transparency in reporting. For further information on Nature Research policies, see our Editorial Policies and the Editorial Policy Checklist.

Statistics

For all statistical analyses, confirm that the following items are present in the figure legend, table legend, main text, or Methods section.

n/a	Confirmed	
☐	☒	The exact sample size (*n*) for each experimental group/condition, given as a discrete number and unit of measurement
☐	☒	A statement on whether measurements were taken from distinct samples or whether the same sample was measured repeatedly
☐	☒	The statistical test(s) used AND whether they are one- or two-sided<br>*Only common tests should be described solely by name; describe more complex techniques in the Methods section.*
☒	☐	A description of all covariates tested
☒	☐	A description of any assumptions or corrections, such as tests of normality and adjustment for multiple comparisons
☐	☒	A full description of the statistical parameters including central tendency (e.g. means) or other basic estimates (e.g. regression coefficient) AND variation (e.g. standard deviation) or associated estimates of uncertainty (e.g. confidence intervals)
☐	☒	For null hypothesis testing, the test statistic (e.g. *F*, *t*, *r*) with confidence intervals, effect sizes, degrees of freedom and *P* value noted<br>*Give P values as exact values whenever suitable.*
☒	☐	For Bayesian analysis, information on the choice of priors and Markov chain Monte Carlo settings
☒	☐	For hierarchical and complex designs, identification of the appropriate level for tests and full reporting of outcomes
☒	☐	Estimates of effect sizes (e.g. Cohen's *d*, Pearson's *r*), indicating how they were calculated

Our web collection on statistics for biologists contains articles on many of the points above.

Software and code

Policy information about availability of computer code

Data collection	Microsoft Office 2016; GraphPad Prism 8.4.2 (GraphPad Software LLC, USA)
Data analysis	GraphPad Prism 8.4.2 (GraphPad Software LLC, USA)

For manuscripts utilizing custom algorithms or software that are central to the research but not yet described in published literature, software must be made available to editors and reviewers. We strongly encourage code deposition in a community repository (e.g. GitHub). See the Nature Research guidelines for submitting code & software for further information.

Data

Policy information about availability of data

All manuscripts must include a data availability statement. This statement should provide the following information, where applicable:

- Accession codes, unique identifiers, or web links for publicly available datasets
- A list of figures that have associated raw data
- A description of any restrictions on data availability

Raw data are associated with Figures:1-6 . Data available on request from the authors.

April 2020

Field-specific reporting

Please select the one below that is the best fit for your research. If you are not sure, read the appropriate sections before making your selection.

☒ Life sciences ☐ Behavioural & social sciences ☐ Ecological, evolutionary & environmental sciences

For a reference copy of the document with all sections, see nature.com/documents/nr-reporting-summary-flat.pdf

Life sciences study design

All studies must disclose on these points even when the disclosure is negative.

Sample size	Not applicable.
Data exclusions	No data were excluded from the analyses.
Replication	The experimental findings were performed in 2 to 11independent experiments as stated at each experiment in the manuscript.
Randomization	Not applicable.
Blinding	Not applicable.

Reporting for specific materials, systems and methods

We require information from authors about some types of materials, experimental systems and methods used in many studies. Here, indicate whether each material, system or method listed is relevant to your study. If you are not sure if a list item applies to your research, read the appropriate section before selecting a response.

Materials & experimental systems

n/a	Involved in the study	
☐	☒	Antibodies
☒	☐	Eukaryotic cell lines
☒	☐	Palaeontology and archaeology
☒	☐	Animals and other organisms
☐	☒	Human research participants
☒	☐	Clinical data
☒	☐	Dual use research of concern

Methods

n/a	Involved in the study	
☒	☐	ChIP-seq
☐	☒	Flow cytometry
☒	☐	MRI-based neuroimaging

Antibodies

Antibodies used	for flow cytometry: anti-human CD19-Pc7 (clone J3-119; Beckman Coulter, USA), anti-human CD27-BV421 (clone M-T271; Becton Dickinson, USA), anti-human IgM-PerCP Cy5.5 (clone MHM-88; BioLegend, USA); anti-human CD19-Pc7 (clone J3-119; Beckman Coulter, USA), anti-human CD27-BV711 (clone O323; BioLegend, USA), anti-human IgM-BV605 (clone MHM-88; BioLegend, USA), anti-human IgA (clone REA1014; Miltenyi, Germany), TT (AJ vaccines, Denmark) biotinylated with EZ-Link Sulfo-NHS-LC-Biotin (Thermo Fisher Scientific, UK) and fluorescently labelled with streptavidin-BV421 (BioLegend, USA)
Validation	multicolor staining with positive and negative controls, see also flow cytometry section

Human research participants

Policy information about studies involving human research participants

Population characteristics	healthy volunteers 18-67 years
Recruitment	individuals that needed to be re-vaccinated were informed about our study
Ethics oversight	The Ethics Committee of the Medical Faculty of the University of Frankfurt

Note that full information on the approval of the study protocol must also be provided in the manuscript.

Flow Cytometry

Plots

Confirm that:

☒ The axis labels state the marker and fluorochrome used (e.g. CD4-FITC).

☒ The axis scales are clearly visible. Include numbers along axes only for bottom left plot of group (a 'group' is an analysis of identical markers).

☒ All plots are contour plots with outliers or pseudocolor plots.

☒ A numerical value for number of cells or percentage (with statistics) is provided.

Methodology

Sample preparation	B cell profile was stained as follows: PBMC were isolated from whole blood or buffy coat by density gradient. Cells were stained with 50µl of mixture of antibodies in PBS with 0.5% FCS for 30 min, 4°C, dark. The cells were resuspend in 100ul of PBS, followed by adding 100ul of 4% PFA (paraformaldehyd) (Sigma-Aldrich Chemie GmbH, Germany) and the suspension was well mixed. The samples were kept in fridge (4°C) over night. Next day the samples were washed before the measurement and cells were resuspended in PBS with 0.5% FCS.<br>For TT enumeration: PBMC were isolated from buffy coat by density gradient. Untouched B cells were enriched from PBMC using the EasySep human B cell enrichment kit (StemCell Technologies, Canada). TT-specific B cells in buffy coat PBMC were stained as described above and measured immediatly without fixing step.
Instrument	BD LSRII SORP flow cytometer (Becton Dickinson, USA); FACS Aria™ Fusion (Beckman Coulter, USA)
Software	Collection of the data: BD FACS Diva software version 8.0.1 (BD Biosciences, Heidelberg, Germany); Analysis: Kaluza Analysis Software (Beckman Coulter, USA)
Cell population abundance	B cells represent 5-10% of whole PBMC
Gating strategy	Gating strategy provided in the manuscript. Positivity or negativy based on unstained controls.

☒ Tick this box to confirm that a figure exemplifying the gating strategy is provided in the Supplementary Information.
